# Revalorization of Almond By-Products for the Design of Novel Functional Foods: An Updated Review

**DOI:** 10.3390/foods10081823

**Published:** 2021-08-06

**Authors:** Pascual Garcia-Perez, Jianbo Xiao, Paulo E. S. Munekata, Jose M. Lorenzo, Francisco J. Barba, Muhammad Shahid Riaz Rajoka, Lillian Barros, Rafael Mascoloti Sprea, Joana S. Amaral, Miguel A. Prieto, Jesus Simal-Gandara

**Affiliations:** 1 Nutrition and Bromatology Group, Department of Analytical and Food Chemistry, Faculty of Food Science and Technology, University of Vigo, Ourense Campus, 32004 Ourense, Spain; pasgarcia@uvigo.es (P.G.-P.); jianbo.xiao@uvigo.es (J.X.); 2 International Research Center for Food Nutrition and Safety, Jiangsu University, Zhenjiang 212013, China; 3 Centro Tecnológico de la Carne de Galicia, Avda. Galicia Nº 4, Parque Tecnológico de Galicia, San Cibrao das Viñas, 32900 Ourense, Spain; paulosochetti@ceteca.net (P.E.S.M.); jmlorenzo@ceteca.net (J.M.L.); 4 Área de Tecnología de los Alimentos, Facultad de Ciencias de Ourense, Universidad de Vigo, 32004 Ourense, Spain; 5 Nutrition and Food Science Area, Preventive Medicine and Public Health, Food Science, Toxicology and Forensic Medicine Department, Faculty of Pharmacy, Universitat de València, Avda, Vicent Andrés Estellés, s/n, Burjassot, 46100 València, Spain; francisco.barba@uv.es; 6 Food and Feed Immunology Group, Laboratory of Animal Food Function, Graduate School of Agricultural Science, Tohoku University, Sendai 980-8572, Japan; shahidrajoka@yahoo.com; 7 Centro de Investigação de Montanha (CIMO), Instituto Politécnico de Bragança, Campus de Santa Apolonia, 5300-253 Bragança, Portugal; lillian@ipb.pt (L.B.); rafael.sprea@gmail.com (R.M.S.); jamaral@ipb.pt (J.S.A.)

**Keywords:** *Prunus dulcis*, almond skins, almond shells, almond hulls, blanching water, waste management, circular economy, food fortification, allergens, sustainability

## Abstract

The search for waste minimization and the valorization of by-products are key to good management and improved sustainability in the food industry. The great production of almonds, based on their high nutritional value as food, especially almond kernels, generates tons of waste yearly. The remaining parts (skin, shell, hulls, etc.) are still little explored, even though they have been used as fuel by burning or as livestock feed. The interest in these by-products has been increasing, as they possess beneficial properties, caused by the presence of different bioactive compounds, and can be used as promising sources of new ingredients for the food, cosmetic and pharmaceutical industry. Additionally, the use of almond by-products is being increasingly applied for the fortification of already-existing food products, but there are some limitations, including the presence of allergens and mycotoxins that harden their applicability. This review focuses on the extraction technologies applied to the valorization of almond by-products for the development of new value-added products that would contribute to the reduction of environmental impact and an improvement in the sustainability and competitiveness of the almond industry.

## 1. Introduction

Almond (*Prunus dulcis* (Mill.) D. A. Webb, *Prunus amygdalus* Batch, or *Amygdalus communis* L.) constitutes the most produced nut worldwide, thanks to its exceptional nutritional composition, including low sugar content, high levels of proteins, unsaturated fatty acids, vitamins, and minerals, as well as health-enhancing phytochemicals [1]. Among the countless almond varieties and cultivars, the Mission, Nonpareil, and California contribute up to 90% of the current worldwide production [2]. The data from the Food and Agriculture Organization (FAO) indicates that over 3 million tons of almond fruits are yearly produced throughout an area of over 2 million ha, being USA (78%), specifically California, Australia (8%), and Spain (6%) the largest producers [3]. Besides fruits, almond production involves the generation of several by-products that are normally discarded, accounting 0.8–1.7 million tons for shells and more than 6 million tons for almond hulls [4,5]. Consequently, the accumulation of almond by-products is causing an increasing concern about their processing, and novel solutions are required to add value to these residues, with the aim of improving the economic profit and environmental sustainability of the large-scale almond production.

### 1.1. Almond By-Products

**Figure 1:**
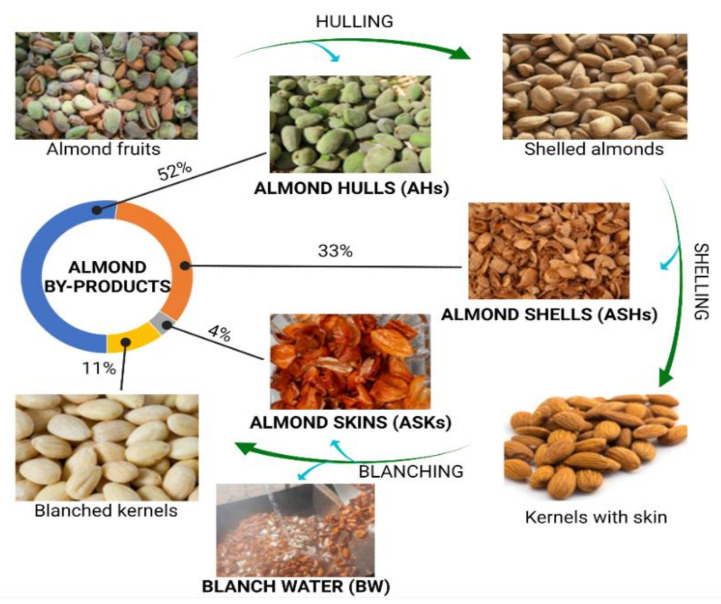
Overall workflow developed during almond production and generation of almond by-products.

Figure 1 shows the common workflow associated with the industrial procedure of almond production. As it is shown, the almond trees are subjected to pruning, thus promoting the generation of branches and leaves that are normally discarded and little attention has been paid to the valorization of these matrices. When drupes ripen, they are harvested and subjected to processing to obtain the almond kernels, or almond meat, which are the major product commercially exploited, involving the generation of the classically known as almond by-products [6]. Harvested drupes enter a first stage of hulling, in which the external coating is removed, forming almond hulls that accounts for the 52% of total produced mass; then, shelled almonds are subjected to shell removal, obtaining the coated almond kernels separated from shells, that represent the 33% of the total fruit. Finally, kernels, which constitutes the 11% of the original almond fruits, are blanched in order to eliminate the skins (which represents the 4%) by a treatment with hot water followed by a final peeling step [7,8]. As a result, four main by-products have gained much attention in the sector of almond production (Figure 1): almond hulls (AHs), almond shells (ASHs), almond skins (ASKs), and blanching water (BW). On the other hand, almond kernels (AKs) are considered as a dense nut with high protein (16%) and lipid contents, the latter representing the 50% of AKs, mostly containing monounsaturated fatty acids (MUFAs, 32.2%), polyunsaturated fatty acids (PUFAs, 12.2%), and a low remaining content of saturated fatty acids (SFAs, <4%) [8]. The rest of nutrients associated to AKs, are represented by a polysaccharidic fraction, containing high fiber and starch proportions, together with a low quantity of simple sugars, and multiple minerals, and vitamins, being an exceptional source of vitamin E (in the form of α-tocopherol) [9,10].

Almond hulls (AHs) refer to the fruit mesocarp of almonds, being the heaviest by-product formed during almond production. They are not destined to nutritional purposes because of their dryness (8–20% of moisture content), leathery texture, and astringency, due to their prolonged exposure to environmental threats [2]. Almond shells (ASHs) constitute the lignocellulosic layer found in the thick endocarp of almond fruits. Due to their woody nature and poor industrial applicability, ASHs are eliminated by incineration or indiscriminate dumped [11]. ASKs is the term used to refer to seedcoat, and are the most explored almond by-product in terms of their content of bioactive compounds, since they are often consumed with the kernel on which they play a paramount role in the antioxidant and antimicrobial defense [2]. BW has been also recently characterized in terms of the bioactive compounds extracted from the kernel during blanching, being legally considered as an industrial waste that should be specially disposed [12]. Among the different applications proposed for the revalorization of almond by-products, such as livestock feeding [13], biofuel production [14], and active carbon formulation [15], a nutritional perspective has been carried out with the aim of characterizing their phytochemical composition, in terms of bioactive compounds production, exploring their suitability as sources of functional ingredients to be applied in the food, pharmaceutical, and cosmetic industries.

### 1.2. Bioactive Compounds from Almond By-Products

**Figure 2:**
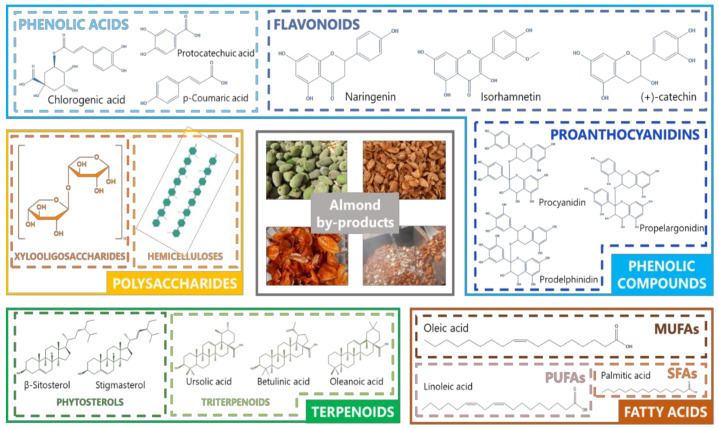
Most representative bioactive compounds found in almond by-products.

Among the different bioactive compounds (BCs) found in almond by-products most reports have been focused on phenolic compounds, including mainly phenolic acids and flavonoids, polysaccharides, terpenoids, and fatty acids (Figure 2). Thus, a recent review by Prgomet et al. [4] widely reported the characterization of almond by-products as a promising source of bioactive compounds, especially phenolic compounds. In addition, these authors review the functional properties of such compounds on the prevention of degenerative diseases. Consequently, an in-depth description of bioactive compounds of almond by-products is required to provide insight about the potential uses of these resources in the field of natural product research.

Phenolic compounds in almond by-products are mainly described by the presence of phenolic acids, flavonoids, anthocyanins, and lignin, among others. In the case of AHs, aqueous extracts of five different Portuguese cultivars indicated that Ferrastar almond presented the highest yields of total phenolic compounds (859.1 mg of gallic acid equivalents (GAE)/g of extract) and flavonoid content [16]. The identification of phenolics from ethanolic extracts of AHs exhibited the presence of hydroxybenzoic and cinnamic acids derivatives and flavan-3-ols and flavonol glycosides [17]. Methanolic extracts of AHs from Nonpareil variety showed that chlorogenic acid and its isomers are the most abundant phenolic compound in this matrix [18]. Such results are in accordance with those of hydroethanolic extracts of different Italian cultivars, being chlorogenic acid the most abundant compound, mostly from Pizzuta hull extracts (4.76 mg/g dry weight, dw), followed by catechin (2.40 mg/g dw) [19]. A recent report has proved that phenolic composition of AHs is highly dependent on the environmental conditions, such as irrigation regimes, showing that hydromethanolic extracts of AHs of Alfândega da Fé cultivar contained high concentrations of naringenin-7-*O*-glycoside (105.5 µg/g dw) and chlorogenic acid (11.6 µg/g dw), together with other phenolic acids in lower amounts, such as protocatechuic and *p*-coumaric acids, and flavonoids, including kaempferol and isorhamnetin glycosides [20]. In the same way, An et al. (2020) [21] revealed that phenolic compounds from acidified hydroethanolic extracts of California AHs are affected by digestion, according to an in vitro gastrointestinal digestion model, showing a 47.8% reduction in total phenolic content in the extracts, that were mainly composed of vanillic acid (115.9 mg/g dw) and 4-hydroxybenzoic acid (35.9 mg/g dw). Concerning phenolic compounds of ASHs, lower contents of these compounds have been reported, as shown by the total phenolic content of methanolic extracts of Iranian almonds, ranging 18.4–122.2 mg GAE/g of extract [22,23]. Such low phenolic contents of ASHs were later assessed, by the absence of both phenolic acids and flavonoids [20], mostly due to its high lignocellulosic composition, with contents of 28.9% lignin and 56.1% polysaccharides, especially celluloses and hemicelluloses, revealing that aliphatic organic acids, 4-hydroxy-5,6-dihydro-(2*H*)-pyran-2-one, and 1,6-anydro-β-D-glucopyranose are the major constituents [24,25]. In this sense, the lignocellulosic nature of ASHs have motivated their use as providers of xylooligosaccharides, which can be effectively adopted as prebiotic and sweetener agents, with a promising potential to be applied as nutraceutical and additive for the food industry [26].

ASKs constitute the most characterized almond by-product, as it has been identified as a prominent source of phenolic compounds, representing a matrix with a content of phenolic compounds around 70–100% of phenolics in the whole almond fruit [27]. Thus, Bolling et al. [28] recently reviewed that flavonoids, phenolic acids, and proanthocyanidins are the main phytoconstituents of ASKs, with estimated values (in mg per g of skin) of 161.0 mg/g flavan-3-ols, 59.8 mg/g flavanones, 493.0 mg/g flavonols, 83.1 mg/g hydroxybenzoic acids, 21.6 mg/g hydroxycinnamic acids, and 69.8 mg/g proanthocyanidins, being (+)-catechin, naringenin 7-*O*-glucoside, isorhamnetin 3-*O*-rutinoside, 5-hydroxybenzoic acid, caffeic acid, and procyanidin B3 + B1 the most abundant compounds of every subfamily, respectively. On this basis, almond phenolics are essentially distributed in the ASKs, accounting for up to 80% of total phenolics on almond fruits [29]. Concerning diversity, 26 phenolic compounds have been differentially identified as a function of solvent employed in their extraction, ranging from methanol to ethanol and aqueous methanol, as determined in the Torito cultivar [12]. Polyphenols from ASKs have been subjected to in vitro human digestion, as well, indicating that phenolic acids are the most bioavailable phenolics, with absorption rates of 68.5% [30]. The phenolic content of ASKs is affected by processing, indicating that blanching and roasting have a negative impact on the phenolic constituents of this matrix. In this sense, BW is obtained after the peeling of almond fruits, and it was also analyzed in terms of phenolic composition, which is obtained by the transference of phenolics from blanched skins. Similar phenolic profiles are found in BW, compared with ASKs, with a special predominance of polar compounds, being naringenin 7-*O*-glucoside, kaempferol 3-O-rutinoside, and catechin the most prevalent compounds [31], together with significant concentrations of proanthocyanidins [32].

Together with phenolic compounds, different phytochemical analyses have pointed at the presence of terpenoids as relevant constituents of almond by-products. Thus, several triterpenoids have been isolated from AHs, mostly represented by ursolic, betulinic, and oleanoic acids and their respective aldehydes, corresponding to <1% of hulls [33]. The betulinic acid has been also reported in the lipophilic extracts of ASHs [24]. In addition to these compounds, later studies revealed the presence of other triterpenoids, such as alphitolic acid derivatives, and corosolic and maslinic acids in the ethylacetate extracts of AHs, together with the phytosterol β-sitosterol [34]. Furthermore, β-sitosterol has been identified as the major sterol of ASKs, accounting for up to 71% of total sterols, whereas stigmasterol has been mostly found in AHs [35], with concentrations about 200 µg/g AH [18]. Additionally, the dichloromethane extracts of ASHs have been reported to contain stigmasterol as the most prevalent phytosterol, followed by β-sitosterol and a little concentration of campesterol [24]. Other nonpolar phytochemicals found in almond by-products are fatty acids, showing ASKs the same fatty acid proportion than AKs, with MUFAs accounting for the 56% of skin lipids (mostly represented by oleic acid), and PUFAs reflecting the 33.6%, mainly by the presence of linoleic acid [31]. Additionally, some SFAs were spotted in ASHs extracts, with a high concentration of palmitic acid [24]. Due to the hydrophobic nature of these compounds, it is supposed to be absent at the BW, although no data on these compounds are currently available.

There is a wide evidence regarding the potential benefits of almond by-products as promising sources of bioactive compounds. The effectiveness of such compounds from almond residues as antioxidant, anti-inflammatory, anticancer, antimicrobial, antiviral, prebiotic, cardioprotective, antidiabetic, and anti-obesity agents have been largely assessed as a result of countless in vitro and in vivo studies, as well as by different interventional clinical trials in humans [35,36]. Thus, almonds together with their by-products can be used as active ingredients for the prevention of chronic diseases, such as cancer, diabetes, and cardiovascular diseases. Due to their precedence from an edible nut, almond by-products can be then applied for the production of new functional foods and nutraceuticals [4]. In this review, a current perspective on the exploitation of almond by-products as sources of bioactive compounds will be proposed, with a special focus on the extraction technologies applied to that end, as well as the description of already-established strategies committed to the fortification of different foods, together with the limitations on the applicability of these by-products. Finally, in order to meet the current global requirements of the food industry concerning environmental consciousness, the exploitation of almond by-products will be incorporated into a sustainability context, exhibiting recent and future strategies aiming at the establishment of a circular economy around the almond industry.

## 2. Extraction Technologies

### 2.1. Conventional Extraction

Conventional extraction (CE) refers to the recovery of bioactive compounds from different matrices using conventional solvents or their mixture, with or without heat or agitation treatment. These techniques are very simple, but in general present low efficiency and high solvent consumption [37]. Maceration, heat-assisted extraction (HAE) and Soxhlet extraction are common examples of CE. Maceration consists of a solid-liquid extraction in which solvent polarity and agitation increase the extraction yield of the target compounds. This technique is simple to operate and does not require expensive and highly specific equipment, however it generally requires long extraction times, a high consumption of solvent and post-treatments, such as filtration and/or centrifugation. Compared to maceration, HAE also involves heating, which further increases the solubility of the compounds of interest in the extraction solvent. Soxhlet extraction works with a continuous heated solvent flow over the sample, which on one hand contributes to obtaining higher yields but on the other can also lead to the degradation of thermolabile compounds as the solvents are used at boiling temperature. As in maceration, Soxhlet needs long periods of time and uses large amounts of solvent and the final extract requires a post-treatment [38].

The use of conventional solid-liquid extraction techniques to extract compounds of interest from almond kernels and almond by-products (hulls, husks, etc.) after their grinding to powder and/or drying, is frequently referred in the bibliography [19,39,40]. Maceration and HAE using low temperatures are frequently adopted approaches for the recovery of phenolic compounds from *P. dulcis* by-products, generally using hydroethanolic solutions (normally ranging ethanol concentrations of 70–80%) and extraction times longer than 1 h, to achieve a higher extraction efficiency (Table 1).

On the other hand, Soxhlet extraction is typically used for the recovery of oil/fatty acids, although it can also be applied for the recovery of phenolic compounds using an appropriate solvent, such as methanol [22]. To obtain the lipidic fraction, petroleum ether is the most used solvent, although hexane is also reported, generally requiring very long extraction times (from 6 to 24 h, Table 1) [22,39,41]. The long extraction times with the associated cost when heat is used and the need of large volumes of solvents, which frequently are toxic and/or with a high environmental impact, are drawbacks that have been driving the change towards the use of innovative and “greener” technologies in the recovery of different bioactive compounds from almonds and its by-products. In this sense, the high amounts of organic solvents and the long extraction times associated with this extraction methodology, together with its scarce selectivity towards target compounds are the major challenges facing its application at an industrial scale.

**Table 1:** Extraction procedures applied to almond by-products for the recovery of bioactive compounds.

Extraction	By-Product	Extraction Procedure	Bioactive Compounds	References
Conventional	AHs	70% EtOH, 50 °C, 6 h	Phenolic acids, catechins	[19]
	AHs	70% EtOH, 24 h	Total phenolic and flavonoid contents	[42]
	AHs	Maceration with EtOAc, 24 h	Total phenolic and flavonoid contents	[43]
	AHs, ASHs	Soxhlet extraction with MeOH, 80 °C, 30 min	Total phenolic content	[22]
	ASHs	HAE with 80% γ-valerolactone,75 mM H_2_SO_4_, 140 °C, 30 min	Lignin and hemicelluloses	[44]
MAE	ASKs	70% EtOH, 2450 MHz, 100 W, 60 s	Flavonol rutinosides	[45]
	ASHs	Choline chloride-oxalic acid, 800 W, 1 min	Lignin	[46]
	AKs	0.5 M NaOH, 2450 MHz, 800 W, 23–67 °C, 3 min	Lignans	[47]
UAE	AHs	51.2% EtOH, 40 kHz, 300 W, 13 min	Phenolic acids, catechin	[19]
	ASKs	Water, 20 kHz, 100 W, 20 min	Phenolic compounds, lipids, proteins	[48]
	ASKs	PEG, 40 kHz, 120 W, 30 min	Proanthocyanidins, chlorogenic acid	[49]
	BW	n.d.	Total phenolic content	[50]
SFE	ASHs	Petroleum ether, 40–60 °C, 90 min, 11 kPa	Holo-cellulose, lignin	[51]
	AKs	Butane, −0.09 MPa, recovery with N_2_ at −4 °C	Total phenolic, phytosterol, tocopherol, and tocotrienol contents	[52]
EAE	ASHs	Endoxylanase from *Thermomyces lanuginosus*, pH 5.5, 50 °C	Xylooligosaccharides	[26]
	ASHs	Cellulase and β-glucosidase, pH 4.85, 48.5 °C	Lignin and cellulose-enriched solids	[53]

Abbreviations: AH: almond hull; AK: almond kernel; ASH: almond shell; ASK: almond skin; EAE: enzyme-assisted extraction; HAE: heat-assisted extraction; MAE: microwave-assisted extraction; n.d.: not defined; PEG: polyethylene glycol; SFE: supercritical fluid-assisted extraction; UAE: ultrasound-assisted extraction.

### 2.2. Microwave-Assisted Extraction

Currently, microwave-assisted extraction (MAE) is one of the most common of all “green extraction methods” [54]. This technology is based on the application of penetrating non-ionizing energy into materials in a spectral frequency of 300–300,000 MHz, resulting in distributed heating due to the molecular friction caused by ionic conduction and the dipolar rotation of polar solvents. The friction and resulting heating can disrupt the cell wall and improves the mass transfer of the compounds of interest into the extracting solvent, reducing extraction times and generally improving the yield when compared with conventional extractions [55,56].

MAE is considered a good alternative at an industrial scale because of its simplicity and reasonably low-cost equipment, with the particularity of allowing the use of different solvents in smaller quantities, reducing energy consumption and time spent on extraction [57]. In addition, this technique can be easily combined with other extraction technologies, such as maceration, HAE, or vacuum extraction, giving rise to the so-called vacuum MAE (VMAE) [58]. On the other hand, it should be noticed that thermo-labile compounds may be degraded due to the heat originating from cavitation, which supposes the greater limitation of this methodology for its large-scale application. Usually, hydroethanolic solutions in different proportions are employed for this extraction methodology, with ethanol being recommended by the U.S. Food and Drug Administration (FDA) as an environmentally non-toxic food grade organic solvent [59,60]. Nevertheless, the use of other solvents like water, hexane, and chloroform have been reported in the literature [61]. The choice of solvent for MAE application is highly important, as it play an essential role on the selectivity towards the target compounds [58].

To date, few studies have reported the use of MAE to recover functional compounds from almond products. Valdés et al. [45] optimized a MAE procedure using a three-factor Box–Behnken design for the extraction of phenolic compounds from ASKs, demonstrating the effectiveness of this method. The use of MAE has also been described for obtaining lignan and lignin from almond and ASHs, respectively, highlighting the use of microwave technology as a fast, efficient and cost-effective technology for lignocellulosic biomass fractionation within a sustainable biorefinery concept [47].

Despite its scarce applicability to almond and by-products thereof, MAE has been successfully applied to the extraction of phenolic compounds from other tree nuts and corresponding by-products such as *Pistacia vera* and *Anacardium occidentale*. In general, the power used in compounds extraction from nuts by-products could reach values ranging from 100 to 1000 W, generally associated with short extraction times (between 1 to 12.5 min), despite it can reach up to 1 h [45,62,63]. Additionally, MAE has been used in other species from the same genus, such as *Prunus armeniaca* L., for protein extraction purposes [62].

### 2.3. Ultrasound-Assisted Extraction

Another method generally classified as “green” is ultrasound-assisted extraction (UAE), being also a promising and widely used method for the extraction of bioactive compounds from plant samples. UAE is also commonly used in the industry due to its affordability, simplicity, ease of scaling-up, and because it offers an efficient alternative to conventional methods as it requires shorter extraction times and lower solvent consumption. In addition, UAE does not require the use of high temperatures commonly associated with other methods, therefore avoiding the degradation of temperature-sensitive compounds [64].

UAE is a solid-liquid extraction method based on acoustic cavitation, where low frequency ultrasonic waves (around 20 kHz) propagate with a high sound power or intensity (higher than 1 W cm^−2^) in the liquid solvent, generating a small-scale intense agitation due to bubble collapse, which facilitates the solvent penetration and affects cell walls integrity, thus enhancing mass transfer into the solvent [65].

Solvent selection in UAE is directly related to the solubility of the compounds of interest, which directly impacts the selectivity of this methodology, as it occurred with MAE. Aqueous solutions of ethanol and methanol are commonly used in this method because they have significantly lower polarity than water, favoring the solubility and diffusion of phenolic compounds [66], nevertheless different solvents, such as sodium hydroxide, hexane, and ethyl acetate can be used [49].

The optimization of UAE can be a challenging process since it requires a detailed understanding of how UAE parameters, such as time, frequency, power, temperature, and solvent-to-matrix ratio influence the treatment results and efficiency [67]. Such optimization is required to meet the operational demands for its application at an industrial level, as UAE presents two major limitations: low efficiency and high operation costs [68]. Among the previous bibliography, most studies report extraction times ranging from 10 to 40 min, which are much shorter as compared to those associated with traditional methods. Polyphenols, phenolic compounds, anthocyanins and proteins are the compounds of interest that are generally recovered from almond by-products using this technique (Table 1) [19,49,50,59].

### 2.4. Supercritical Fluid-Assisted Extraction

Supercritical fluid technology uses the distinct properties of a solvent in their supercritical state, being a commonly used technique by the pharmaceutical industry, since the solubility of solid in a supercritical fluid can be 3–10 folds magnitude higher than in the liquid state [69]. Moreover, the supercritical fluid can be used either as an effective solvent, by dissolving compounds within its phase, or as an anti-solvent when it is committed to precipitating the solute [70].

Carbon dioxide (CO_2_) is the supercritical fluid most used in food industries. This recurrent use is due to the fact that CO_2_ is chemically inert, non-toxic, cheap and considered a food grade and Generally Recognized As Safe (GRAS) solvent. However, due to the low affinity of polar compounds, co-solvents such as ethanol and methanol have been used to enhance the solubility of these molecules aiming at increasing the performance and selectivity of this extraction method [71]. Moreover, besides supercritical fluid-assisted extraction (SFE), pressurized liquid extraction has been proposed as an alternative to the compressed fluid-assisted extraction of polar compounds from natural matrices, although its use on almond by-products has not been reported, to date [72].

Supercritical fluid-assisted extraction (SFE) is considered a good alternative to conventional solvent extraction. Almond oils obtained via CO_2_ supercritical fluid extraction are of high quality, however when compared to conventional solvent extraction, SFE involves the use of more expensive equipment, although the final product does not need any further treatment [73,74]. According to the literature, SFE is mostly used to extract the oil from almond kernels. Compared to conventional methods, it allows the extraction of higher yields of bioactive compounds, thus resulting on a tocopherol-enriched and/or polyphenol-enriched almond oil. Moreover, the use of CO_2_ as a solvent in the supercritical extraction of almond oil has several advantages over conventional solid-liquid extractions, namely avoiding the use of harmful and toxic organic solvent, not needing to refine the obtained oil, and the higher yield obtained by this method [52,53,75]. Therefore, in the last years, SFE is attracting the industry’s attention as consumers are increasingly interested in products obtained with cleaner technologies as an alternative to the employment of organic solvents. Nevertheless, SFE, as a novel extraction technology, presents relevant limitations as it is its high cost and the difficulty to carry out serial continuous extraction [68], which require further optimization for its scaling-up at industrial level.

### 2.5. Enzyme-Assisted Extraction

Enzyme-assisted extraction (EAE) is a solid-liquid extraction based on enzymatic reactions. This technique is a potential alternative to conventional solvent-based extractions due their efficiency, being also a sustainable and eco-friendly approach. However, the high cost attributed to enzymes is a challenge to deploy this technique at industrial scale. Among the benefits associated with EAE, the enzymatic catalysis promotes the development of high-level specificity extractions, providing a prominent selectivity to EAE [76,77,78]. In addition, the ability of enzymes to degrade cell walls and membranes enables reaching improved release of compounds and thus, more efficient extractions. The combination of factors, such as enzyme concentration, pH, temperature, solvent, and time is essential to obtain the highest hydrolytic activity and should be considered in during EAE optimization. As well, the combination of enzymes is frequently assayed to increase extraction efficiency [79]. In this way, the wide range of factors affecting EAE requires the implementation of monitoring systems to control enzymatic efficiency, making its application at industrial scale difficult together with the high cost associated with enzyme production [80].

Different compounds of interest can be obtained through EAE by varying the type of process and enzyme. In this sense, the valorization of almond by-products by this approach contributes to the circular economy of this agricultural sector and contributes to the achievement of the goal of zero waste while increasing the economic value of almond’s by-products. Souza et al. (2020) [81] evaluated the effect of EAE on the recovered oil and protein from almond cake and concluded that it enabled a similar extractability while resulting in a creamy fraction easier to be demulsified (corresponding to an oil recovery of 99%), together with a skim fraction rich in proteins with lower surface hydrophobicity and higher solubility at lower pH, which can be particularly important for applications in acidic media. Morales et al. (2020) [53] proposed the integral valorization of ASHs through a biorefinery approach in which enzymatic hydrolysis was used to obtain glucose and generate solids rich in lignin and cellulose. In other work, Singh et al. (2019) [26] reported the application of EAE to almond bark for recovering low-degree polymerized xylooligosaccharides.

## 3. Food Fortification Using Almond By-Products

The incorporation of foods with almond shell, hull and skin or their extracts is shown in Table 2. Recent studies have explored both the use of almond by-products as feedstock to produce foods of animal origin and their incorporation as ingredients in foods. Regarding the incorporation into animal feedstock, a recent study with milk cows indicated that AHs (7–20% in feeding) caused a significant effect in the content of milk fat and protein contents [13]. Linear correlations were obtained between these macronutrients and the level of AHs in feeding: fat content and protein content decreased with the increasing levels of AHs in cow feeding, thus suggesting an enhancing nutritional potential associated with these almond by-products. These effects were attributed to the metabolism of carbohydrates and proteins in the rumen, according to the authors. A related experiment with AHs reported contrasting results in terms of milk composition [82]. In this case, the incorporation of AHs did not affect fat, protein, or lactose content. Moreover, no effect in terms of fatty acids were observed between the milk samples obtained from cow in control and almond hull diet. As a result, the influence of AHs as feedstock remains unclear and further investigations are required on this concern.

Besides feedstock, almond by-products have been recently explored in the basis of ingredients for the fortification of food products. In this way, the potential fortification of eggs with AHs was studied in a recent study [83]. In this case, AHs were added to the feeding of laying hens at two levels: 7.5 and 15%. According to these authors, no significant effects were reported in terms of egg quality (percentages of yolk, albumen, and shell, Haugh unit, specific gravity, and egg size). Interestingly, the authors reported that hens in the supplemented group displayed a reduction in fat and lean mass in relation to animals in non-supplemented group.

Another interesting experiment was recently carried out to effect of AHs and ASHs as a bed of feedstock in the production of edible black soldier fly (*Hermetia illucens* L.) larvae [84]. In this case, the use of almond by-products bed in combination with aeration increased the content of calcium by 18% in the insects. Additionally, the group of larvae reared in almond by-products beds with high aeration rate in the bioreactor had higher harvest weight and yield (three and five times higher, respectively) than those produced using a bed with low aeration rate.

The incorporation of almond by-products as fortifying ingredients has also been showing promising results. For instance, the addition of an alkali extract rich in dietary fiber from ASHs (15.3%) increased the content of total, soluble, and insoluble dietary fiber of biscuits [85]. In addition, the use of these high-fiber ingredients also affected the instrumental and sensory scores of colors and hardness of biscuits. No significant effects were reported for flavor, crispness, mouthfeel, hardness, and overall acceptance between control and fortified biscuits. It is also important to mention that this study explored the combinatory effect of almond extract with stevia (a natural sweetener), as well. This combination had a similar effect in terms of dietary fiber enhancement to the sole addition of alkali extract but led to a significant reduction in the sensory properties of almond-enriched biscuits, especially at 14.8% of alkali extract. Additionally, a related experiment indicated that ASKs can also improve the technological properties of wheat flour dough in a concentration dependent effect (from 30 to 100 g/kg), which supports its incorporation into bakery products [50].

These studies indicate that almond by-products, mostly hulls and skins, can be a seen as a multicomponent source of nutritionally relevant compounds for food fortification for the incorporation either in animal feeding or in the use as food ingredient in processed foods. However, more studies are necessary to improve the knowledge about the factors limiting the fortification of food with almond by-products, especially in animal feedstock studies.

## 4. Limitations on the Applicability of Almond By-Products

### 4.1. Allergens

According to the literature, the incidence of nut allergy in the general population is 1%. Almonds are nuts that are commonly consumed worldwide, and their consumption constitutes a potential allergen risk [86]. Indeed, the allergens from almonds are the third most common reported nuts allergens in the United States of America behind cashew nut and walnut [87]. Thus, numerous food allergens have been characterized from eight native almonds on the basis of their biochemical functions (Table 3). Amongst them, there are four almond-derived allergens officially reported as food allergens, according to the World Health Organization (WHO)–International Union of Immunologist Societies (IUIS) list, namely: Pru du 3, Pru du 4, Pru du 5, and Pru du 6 [88,89,90,91,92].

In general, almond-mediated allergies are usually associated with poplar pollen allergies from other fruits of Rosaceae family members. In most of the cases, immune reactions are mild with a prominent clinical manifestation related to oral allergy syndrome [93,94]. Pru du 6, also known as amandin, is one of the firstly described allergens from almonds, as well as the major almond allergen, accounting for approximately 65% of total almond proteins, so it can induce a severe immune reaction upon ingestion [95]. In 2009, Pru du 3 (non-specific lipid transfer protein) was added into the WHO-IUIS allergen database [96]. In 2006, Pru du 4 was classified as a food allergen and added into the WHO-IUIS allergen database, as well, after the conduction of a study in which more than 40% of participants developed clinical symptoms derived from this allergen consumption [91]. In short, almond is largely used by the food industry due to its flavor, nutrients, and numerous health benefits despite the development of several allergic reactions. In this sense, little efforts have been made on the study and characterization of these allergens, in comparison with other nut-derived allergens, such as those of peanuts and other tree nuts. Therefore, there is an urgent need to explore the potential allergens proceeding from almond, along with their proper nomenclature and the definition of their structure-function relationships.

### 4.2. Mycotoxins

As essential economical resources of several regions worldwide, including California and Mediterranean countries, almond production is constantly subjected to several studies focused on the mycotoxin contamination, being one of the leading problems behind production and quality losses in this sector. The most prominent fungal contaminations reported on almonds are those caused by different strains belonging to *Aspergillus*, *Eurotium*, *Penicillium*, and *Rhizopus*. Much attention has been paid to both *Eurotium* spp. and *Aspergillus* spp. contaminations, causing a severe negative impact on all the stages of almond production cycle, from field harvesting to storage and market production [97,98]. Thus, among the several problems associated with fungal contaminations, aflatoxins produced by different *Aspergillus* species constitute one of the major threats to almond production, which may proliferate under insufficient storage and handling conditions [99].

Aflatoxins are difuranocoumarin isomers, named as AFB1, AFB2, AFG1 and AFG2, differentiated by their fluorescent properties [100]. These compounds may cause severe toxicity and carcinogenicity in numerous animals as well as in humans, in which their consumption leads to a systemic symptomatology collectively known as aflatoxicoses, characterized by hemorrhages, acute liver damage, edema, digestive disorders, malabsorption, and, eventually, death [101]. Hence, fungal contamination is a major problem in almond processing. Since the full eradication of fungal contamination is a hard goal to achieve, understanding the occurrence and diversity of fungal population and their mycotoxigenic potential, as well as improving the environmental and storage conditions, will greatly contribute to reduce the risk of contamination.

### 4.3. Cyanogenic Compounds

Cyanogenic compounds (CCs) occur in a wide range of plant species. The potential toxicity attributed to CCs is due to enzyme-mediated hydrogen cyanide (HCN) production, which may cause acute cyanide poisoning and participate in the pathogenesis of multiple chronic diseases [102]. It was estimated that from the almost 25 CCs currently known, a high proportion is derived from the edible part of almonds, sorghum, and bamboo shoots, providing a negative bitter taste [103].

Amygdalin, commonly known as B17 vitamin, was isolated from almond seeds. As a naturally occurring CC considered as a vitamin, this compound has shown numerous effects for the treatment of chronic diseases, such as cancer and asthma [104]. Nevertheless, amygdalin may be susceptible to glycosylation, and undergo a metabolic degradation to glucose and mandelonitrile, which ultimately leads to HCN generation [105].

HCN has been widely identified as a toxic substance to human health, developing several mechanisms of action of different nature, including cytotoxic effect via the induction of apoptosis-mediated cell signaling pathways and the inhibition of cell respiration by the cytochrome oxidase blockage [106,107,108,109]. On these bases, the WHO, the European Food Safety Agency (EFSA), the Environmental Protection Agency (EPA) suggested that the amygdalin daily oral dose for adult humans (50–60 kg) should be in the range of 0.6–0.72 mg, 0.18–0.22 mg and 6–7.2 mg, respectively [110]. Furthermore, the ingestion of 500 mg of amygdalin was predicted to produce 180 mg of HCN, approximately, which is supposed to be lethal for humans [111]. Table 4 shows an overview of the toxigenic effects derived from amygdalin consumption in patients administered with different HCN dosage.

## 5. Sustainability and Future Perspectives for Almond Revalorization

**Figure 3:**
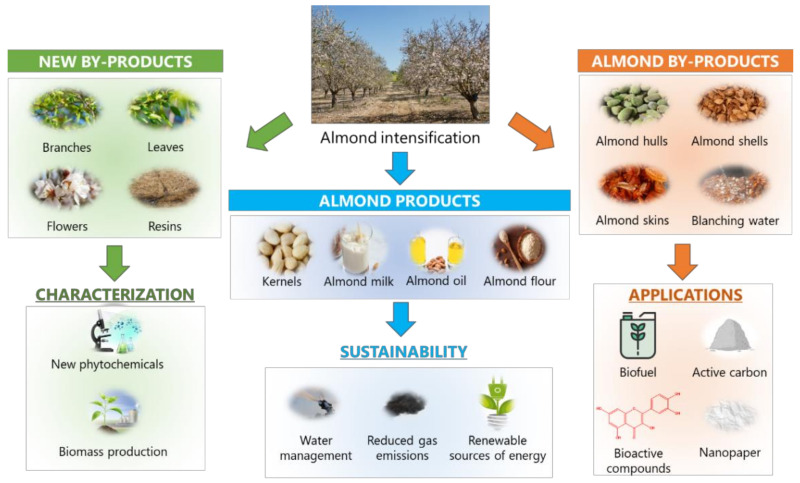
Future perspectives regarding the valorization of almond products and by-products.

As stated throughout this review, almond by-products can be incorporated in a wide range of foods because of their nutritional and phytochemical composition. Almond intensification (Figure 3) provides a wide range of products, mainly focused on the food and cosmetic industries, including kernels, milk, oil, and flour. In the same way by-products have been exploited by means of different applications. Intense efforts are being developed with the aim of achieving a sustainable exploitation of the great amounts of waste generated during almond production, in order to establish a solid circular economy system around this sector.

Besides the multifaceted applications of almond by-products, other strategies should be taken into account for the accomplishment of the sustainable goals proposed for this industry. Given the fact that the bioactive compounds from almond and its by-products are highly influenced by environmental conditions, rational approaches should be developed to increase the biosynthesis of these compounds. With the aim of promoting an environmental-friendly stimulation of bioactive compounds production, different authors have already revealed that irrigation regime plays a dual role on almond production, since water constitutes the main resource employed during almond production, accounting for the 45.8% of the environmental impact associated with this procedure [3]: on one hand, sustained deficit irrigation constitutes a paramount strategy to ensure a proper water management in wide areas involved in almond production, as it the case of semi-arid Mediterranean regions [117]; on the other hand, such limited water regime may drive to an improvement of physical and sensory parameters of almond fruits, as well as increasing the biosynthesis of bioactive compounds (especially naringenin 7-*O*-glucoside and isorhamnetin-3-*O*-rutinoside), as already reported for AHs, which exhibited an increase in the irrigation-mediated production of phenolic compounds, and ASKs, mostly influenced by the agro-climatic conditions [20]. As a result, water management has been revealed as a critical factor related to the sustainability of almond production, with a higher significance in arid regions, which represent the most prevalent production areas worldwide.

In addition to water management, almond production must face additional issues concerning sustainable intensification, as it the case of greenhouse gases (GHG) emission. In detail, GHG production has been estimated, indicating that the production of 1 kg of raw almond and by-products provokes the emission of 1.6 kg of carbon dioxide equivalents (CO_2eq_) [118]. In order to counter almond production-associated pollution, almond by-products, mostly ASHs, have been reported as biobased heat and energy sources, and recently suggested to be subjected to scaled-up bioreactors for its large-scale production [119]. In fact, the feasibility of such application has been already assessed, showing a carbon conversion efficiency of 75%, as recently proved by Kaur et al. (2020) [14]. AHs have been also regarded as a potential source of biofuels, through the recently coined concept of “almond refinery”, in which hulls are subjected to hydrothermal treatment to simultaneously synthesize biofuels and valuable compounds [120].

Additional applications facing the revalorization of almond by-products, with the aim of preventing their environmental impact, include the production of activated carbons (ACs). Such approach presents a dual benefit: from the point of view of waste management, ASHs have been largely employed for the production of ACs, by CO_2_ gasification [121]. In this sense, the CO_2_ emitted during almond production could be re-directed to that aim. The adsorptive properties attributed to ACs can give rise to a sustainable strategy, by which heavy metals (lead, copper, etc.) and other contaminants from industrial wastewaters can be efficiently removed [122]. This biobased adsorptive matrix has been also reported to be an excellent purification agent, promoting the selective isolation of phenolic compounds from complex plant extracts, thus supposing an efficient and green technique for the production of bioactive compounds [123]. Due to the lignocellulosic nature of ASHs, this almond by-product has been also exploited as a source of pure cellulose for the production of nanofibers that can be used in the manufacturing of nano papers, as a result of their delignification [124].

On top of the well-established almond by-products, largely reported for their valuable applications, the sustainability of almond production can be still improved and optimized. There are some under-explored residues, produced during almond production, which require much attention regarding the exploitation as profitable resources. In this way, as it occurred with ASHs, the lignocellulosic profile of almond tree bark and branches from pruning have been recently assessed as effective natural sources of ACs, promoting the removal of highly contaminant synthetic dyes from wastewater [15], as well as a source of biofuel via wet torrefaction [125]. Moreover, almond tree leaves and flowers are another side by-products obtained from pruning that should be also subjected to re-valorization, especially in terms of bioactive compounds production, as it occurs with olive tree leaves, and biomass collection to produce green energy.

## 6. Concluding Remarks

In this review, the most recent advances on the exploitation of almond by-products were thoroughly addressed, which represent thousands of tons of waste that are yearly produced as a side-effect of almond production. Among by-products, almond skins and the blanching water have been identified and considered as sources of bioactive compounds, specifically phenolics, triterpenoids, and MUFAs. Thus, such compounds can be potentially used as food additives, being largely exploited in the food industry, besides the classical food products derived from almond intensification, including almond kernels (also known as almond meat), flour, oil, and milk. Due to the health-enhancing properties of such bioactive properties, novel extraction methodologies should be developed to ensure their sustainable purification, employing green strategies, as it is the case of MAE, UAE, and SFE. The corresponding bioactivities of these compounds are of high interest in the fortification of different food products, giving rise to the production of functional foods with enhanced quality properties. Additionally, other by-products can be used as sources of sustainable resources, in particular ASHs, enhancing the profitability associated with almond industry, promoting their application as CO_2_-based active carbons and biofuel production. These applications may facilitate the establishment of a circular economy around almond industry, by the re-introduction of by-products derivatives into the productive system, as it is the case of biofuel and active carbons, that may assist in the purification of compounds of interest and in the removal of contaminants form wastewaters at the same time. Additionally, novel resources usually underexplored, such as almond tree leaves, flowers, and resins, should be also explored facing their valorization, as it occurs in other industries, including the olive oil and wine productive sectors.

## Figures and Tables

**Table 2:** Fortification of food products with almond by-products.

Almond By-Products	Administration	Fortified Food	Nutritional and Technological Effects	References
AHs	7–20% in cow feedstock	Milk	Increased fat content, reduced protein content, no effect in lactose and total solids	[13]
	4.0 kg of dry matter/day in cow feedstock	Milk	No effect in fat, protein and lactose contents or fatty acids	[82]
	7.5 and 15% in laying hens feedstock	Eggs	No effect in egg quality	[83]
AHs and ASHs	Bed of feedstock	Edible larvae	Increased harvest weight, harvest yield and calcium content	[84]
ASHs	3–15.3% alkali extract	Biscuit	Increased TDF, SDF and IDF, a* value, and hardness; reduced L* and b* values, sensory scores for color; no effect in sensory scores for flavor, crispness, mouthfeel, hardness, and overall acceptance	[85]
ASKs	(30–100 g/kg)	Wheat flour dough	Increased water absorption, dough stability, tenacity/extensibility ratio, L* and a* value; reduced softening index, deformation energy, b* value; no effect in dough development time	[50]

Abbreviations: a*: greenness-redness; AH: almond hull; AK: almond kernel; ASH: almond shell; ASK: almond skin; b*: blueness-yellowness; IDF: insoluble dietary fiber; L*: lightness; SDF: soluble dietary fiber; TDF: total dietary fiber.

**Table 3:** Almond-derived food allergens and their clinical effects.

Allergens	Mw	Biochemical Functions	Effects on Food Processing	Clinical Effects
Pru du (γ-conglutin)	45 kDa	Vicillin storage protein	n.d.	Unclear symptoms
Pru du 1	17 kDa	Protection against pathogens and environmental stresses	Wet heat protection	Reduction of immunoglobulin E (IgE)-mediated reactivity. Mild immune reaction
Pru du 2	23–27 kDa	Protection against pathogens and osmotic stresses	Heat and pH protection	Potent immunogenicity
Pru du 2S	12 kDa	Nut storage protein	Heat resistance	Unclear symptoms
Pru du 3	9 kDa	Lipid transfer protein	Heat and pH protection	Systemic and life-threatening symptoms
Pru du 4	14 kDa	Actin-binding protein	Heat dissipation	Mild immune response
Pru du 5	10 kDa	Involvement in protein synthesis	Thermal stability	IgE-mediated allergic reactions
Pru du 6 (amandin)	360 kDa	Storage protein	Thermal stability	Severe IgE-mediated allergic reactions

Abbreviations: IgE: immunoglobulin E; Mw: molecular weight; n.d.: not determined.

**Table 4:** Toxigenic effects of amygdalin upon oral administration.

Research Model	Dose ^1^	Toxic Events	References
58-year-old healthy woman	50 bitter almonds	Dizziness, vomit, encephalopathy, severe lactic acidosis	[112]
4-year-old male child with malignant brain disease	2000 mg/day	Severe metabolic and lactic acidosis, unresponsiveness	[113]
41-year-old healthy woman	15 g	Metabolic acidosis, respiratory insufficiency, hypothermia	[114]
35-year-old mentally ill woman	>20 almonds	Fast apnea, hypoxia, and respiratory insufficiency	[115]
57-year-old woman with breast cancer	Overdose of amygdalin	Death, HCN accumulation of 218 µg/dL	[116]

^1^ Dose refers to amygdalin dosage unless otherwise stated.

## Data Availability

Not applicable.
